# Dexmedetomidine Protects against Transient Global Cerebral Ischemia/Reperfusion Induced Oxidative Stress and Inflammation in Diabetic Rats

**DOI:** 10.1371/journal.pone.0151620

**Published:** 2016-03-16

**Authors:** Xianzhang Zeng, Honglei Wang, Xichun Xing, Qi Wang, Wenzhi Li

**Affiliations:** Department of Anaesthesiology, Second Affiliated Hospital of Harbin Medical University, Harbin, Heilongjiang, China; Indian Institute of Integrative Medicine, INDIA

## Abstract

**Background:**

Transient global cerebral ischemia/reperfusion (I/R) is a major perioperative complication, and diabetes increases the response of oxidative stress and inflammation induced by I/R. The objective of this study was to determine the protective effect of dexmedetomidine against transient global cerebral ischemia/reperfusion induced oxidative stress and inflammation in diabetic rats.

**Methods:**

Sixty-four rats were assigned into four experimental groups: normoglycemia, normoglycemia + dexmedetomidine, hyperglycemia, and hyperglycemia + dexmedetomidine and all subsequent neurological examinations were evaluated by a blinded observer. Damage to the brain was histologically assessed using the TUNEL staining method while western blotting was used to investigate changes in the expression levels of apoptosis-related proteins as well as the microglia marker, ionized calcium-binding adapter molecule 1 (Iba1). Water content in the brain was also analyzed. In addition, hippocampal concentrations of malondialdehyde (MDA) and Nox2 (a member of the Nox family of NADPH oxidases), and the activity of superoxide dismutase and catalase were analyzed. Finally, changes in serum concentrations of tumor necrosis factor-α (TNF-α) and interleukin (IL)-6 were detected.

**Results:**

Results showed that diabetes increased brain water content, the number of apoptotic neurons, early neurological deficit scores, oxidative stress (MDA and Nox2) and inflammation (pro-inflammatory cytokines including TNF-α and IL-6) levels following transient global I/R injury, but that these symptoms were attenuated following administration of dexmedetomidine.

**Conclusions:**

These findings suggest that dexmedetomidine can significantly alleviate damage resulting from I/R, and this mechanism may be related to a reduction in both oxidative stress and inflammation which is normally associated with I/R.

## Introduction

Transient global cerebral ischemia/reperfusion (I/R) is a major perioperative complication. With aging of the surgical population in western countries and rapid development in China [1,2], an increasing frequency of perioperative cerebral I/R injury is predicted. During this pathological process, increased oxidative stress and inflammation may play an important role and lead to cellular and subsequent tissue damage [3].

Diabetes is an important risk factor for ischemic stroke [4] and is known to increase the oxidative stress and inflammation response induced by I/R [5]. Patients with diabetes who have ischemic strokes and intracerebral hemorrhages have higher mortality rates than non-diabetic patients [6] and are more likely to have secondary ischemic events and organ damage. Animal experimental models of transient ischemia have also found that diabetic hyperglycemic animals frequently develop post-ischemic seizures and that streptozocin (STZ)-induced hyperglycemia resulted in exacerbated post-ischemic brain damage [7, 8]. Thus, there is a need to investigate protective methods for diabetic patients who suffer a cerebral ischemic event.

Both *in vivo* and *in vitro* studies have demonstrated that the α_2_ adrenergic receptor agonist, dexmedetomidine, has protective effect against I/R injury in the heart, kidney, intestines and testis in normoglycemic animal models [9–12] and recent studies have found the same protective effects were evident in the heart and lungs of hyperglycemic rats [13, 14]. A strong body of evidence exists that demonstrates the neuroprotective effects of dexmedetomidine in normoglycemic animal models [15–19]. Yet to date, there is little published data addressing the effects of dexmedetomidine in animal models of diabetes. The objective of this study was to determine whether dexmedetomidine may have a neuroprotective effect on STZ-induced 4-week diabetic rats after transient global cerebral ischemia.

## Materials and Methods

### Animals

Sixty-four male, 8-week-old Sprague-Dawley rats (280–320g) were procured from the Central Animal House of Harbin Medical University in Harbin, China and the protocol for experimentation described in this article was approved by the Animal Committee of Harbin Medical University. Rats were kept in cages (three in every cage) under controlled conditions (21 ± 2°C, 12h light ⁄ 12h dark cycle) and had free access to food and water before the experiment.

### Experimental protocol

Sixty-four rats were randomly assigned into two major groups according to the blood glucose levels of each rat: normoglycemic rats (NG, n = 32) and diabetic hyperglycemic rats (HG, n = 32). Animals in each group were randomly subdivided into two groups according to dexmedetomidine administration. Therefore, there were four groups in this study (normoglycemia (NGC), normoglycemia + dexmedetomidine (NGD), hyperglycemia (HGC), and hyperglycemia + dexmedetomidine (HGD); n = 16 for all groups). Within the control groups (NGC and HGC), rats were administered saline intravenously for 90min at a rate of 5 mL/kg/h 30min prior to the onset of ischemia. The NGD and HGD groups received dexmedetomidine intravenously for 90 min at a rate of 5 μg/kg/h/5mL (total dose 7.5 μg/kg) 30min prior to the onset of ischemia.

**Fig 1:**
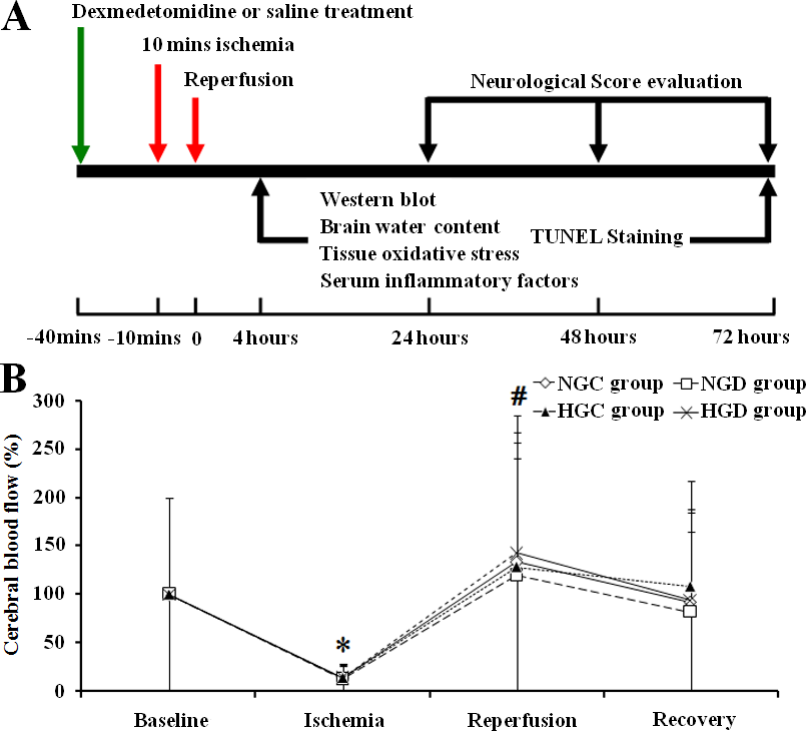
A, Experimental timeline for ischemia, reperfusion, neurological score evaluation, TUNEL staining, western blotting, determination of brain water content, tissue oxidative stress, and serum inflammatory factors. B, Effect of dexmedetomidine on CBF before, during, and after cerebral ischemia in normal and diabetic rats. * *p* < 0.05, ischemia vs baseline within all groups; # *p* < 0.05 reperfusion vs ischemia within all groups.

Neurological examination for each group was evaluated by a blinded observer every 24h for a period of 3 days. Following neurological evaluation, the rats were sacrificed and brains were quickly harvested for terminal deoxynucleotidyl transferase-mediated dUTP-biotin nick end labeling assay (TUNEL) staining. In addition, a subset of rats underwent reperfusion and after 4h were sacrificed and brains were harvested for western blotting, oxidative stress and brain water content examination. Blood was also drawn for serum concentrations of cytocaine at this time point. (See **Fig 1A** for experimental protocol).

### Induction of diabetes

After 12h fasting, the rats were injected intravenously through the tail vein with a single dose of 45mg/kg STZ freshly dissolved in 0.1 M citrate buffer (pH 4.5). STZ-treated animals were allowed to drink 5% glucose solution during the first 24h in order to induce hypoglycemic shock. Diabetes in rats was observed by moderate polydipsia and marked polyurea and random blood glucose levels were estimated 48h after STZ injection. Animals having a blood glucose level of more than 12mmol/L were selected for the study according to previous observations regarding extensive neuronal necrosis in diabetic animals [20]. Age-matched rats were injected with an equal volume of citrate buffer to serve as nondiabetic normoglycemic controls. These rats were used 4 weeks after STZ or citrate buffer injections. Prior to the induction of ischemia, diabetic rats were not given any food but had free access to drinking water.

### Preparation of ischemia

The operative procedures were conducted according to previous studies [21, 22]. Briefly, rats were anesthetized with 4% isoflurane (ICS Chemicals, England). Thereafter, the animals were endotracheally intubated and connected to a respirator for ventilation and the isoflurane concentration was lowered to 2% for the operative period. The common carotid arteries were isolated via a middle neck incision and encircled with sutures for later clamping. A tail artery and vein, and a femoral vein were cannulated for blood pressure monitoring, blood sampling, and drug infusion. All incisions were infiltrated with 0.25% bupivacaine. Rectal and skull temperatures were both maintained at 37°C by a combination of a homoeothermic blanket control unit and a heating lamp.

### Induction of Ischemia

Before the ischemia induction, isoflurane was reduced to 0.8%–1.0% and the fentanyl (10 μg/kg) was administrated intravenously, followed by a continuous infusion at 25 μg/kg/h. Forebrain ischemia lasting 10min was induced in both diabetic and non-diabetic rats by clamping the bilateral common carotid arteries and bleeding hypotension to 35 ± 2mmHg. Reperfusion was introduced by reinfusing the shed blood over 10min and by releasing the clamps placed around the carotid arteries. Animals were killed after 4h or 3 days of reperfusion.

### Cerebral blood flow Measurement

Cerebral blood flow (CBF) was measured with a laser Doppler flowmeter (PeriFlux System 5000, Perimed AB, China). After incision of the scalp, a non-penetrating burr hole was drilled into the cranium (from bregma: anteroposterior [AP] -3.8mm, mediolateral [ML] 2.0mm) and a probe was attached to the skull with a plug. CBF was measured 5min before bleeding induction (the baseline value), at the time of ischemia, and every 5min after reperfusion. CBF values were expressed as percentages relative to baseline (100%).

### Neurological evaluation

After reperfusion, rats were transferred to individual cages and allowed free access to food and water. Neurological examination for each group (n = 16 for each group, respectively) was evaluated by a blinded observer every 24h for a period of 3 days, starting 24h after the ischemic event, using the neurological deficit score for rats as described previously [23, 24]. The average daily scores for 3 days could range from 0 (no dysfunction) to 100 (worst dysfunction).

### Brain histopathology and TUNEL staining

After neurological evaluation, rats were anesthetized and transcardial perfusions were performed (100 mL isotonic saline, followed by 100 mL 4% paraformaldehyde solution at a pressure of about 100 cm H_2_O). Brains were removed and stored in paraformaldehyde for subsequent histopathologic examination. The forebrain was dissected into coronal blocks and embedded in paraffin, and 6 mm sections were cut and mounted on slides. The sections (n = 8 for each group) were treated an *in situ* cell death detection kit according to the manufacturer’s instructions (Roche, Indianapolis, IN, USA). Diaminobenzidine was used as a chromogen, and TUNEL-positive apoptotic cells exhibited brown nuclear cytoplasmic staining. The number of apoptotic cells in the hippocampus and cortex in the parietal lobe was semi quantitatively assessed using light microscopy (five different random high-powered fields/sector, 400x magnification). All TUNEL-positive cells were counted and the numbers of apoptotic cells were calculated for each area, respectively. All experimental analyzes were done blindly.

### Western blot analysis for apoptosis-related proteins and Iba1

Apoptosis-related proteins, Bcl-2 (Mito), Bax (Mito), cytochrome c (Cyto) and the microglia marker, ionized calcium-binding adapter molecule 1 (Iba1, Cyto), were measured by western blot analysis (n = 5). Briefly, 4h after reperfusion left hippocampal tissue was obtained and stored at -80°C until further analysis. Whole-cell lysates were obtained by homogenizing the tissue with a homogenizer (Bullet Blender Homogenizer, Next Advance, Inc, Averill Park, NY, USA) in a 5-mL volume of buffer (20 mmol/L HEPES, 1.5 mmol/L MgCl_2_, 10 mmol/L KCl, 1 mmol/L EDTA, 1 mmol/L EGTA, 250 mmol/L sucrose, 0.1 mmol/L phenylmethylsulfonylfluoride, 1 mmol/L dithiothreitol, and protease inhibitor cocktail tablets; pH 7.9). Samples were further centrifuged at 800×*g* for 15min at 4°C to separate the sample into supernatant A and pellet A. Supernatant A containing cytosolic/mitochondrial proteins was further centrifuged at 16,000×*g* for 30min at 4°C to separate supernatant B from pellet B. Supernatant B was used as the cytosolic fraction, and pellet B was used as the mitochondrial fraction after resuspension in buffer. The protein concentrations of samples were determined using the Pierce bicinchoninic acid Protein Assay kit (Thermo Scientific, West Palm Beach, FL, USA) with a bovine serum albumin standard. Equal amounts of protein were separated on 10% NuPAGE Novex Bis-Tris gradient gels (Invitrogen, Grand Island, NY, USA) and transferred to nitrocellulose membranes (Invitrogen). After blocking in 5% nonfat milk for 1 h at room temperature, membranes were incubated with respective primary antibodies specific for Bcl-2 (Mito), Bax (Mito), cytochrome c (Cyto), Iba1 (Cyto) and β-actin (all at 1:1000, Cell Signaling Technology Inc., Danvers, MA, USA) overnight at 4°C, followed by incubation with secondary antibodies for 1 h at room temperature. Finally, the membranes were scanned with an Odyssey infrared imaging system (Licor Odyssey).

### Brain Water Content

The left parietal lobes from eight rats in each group were weighed immediately (wet weight) 4h after reperfusion, then stored at 70°C for 7 days before being weighed again (dry weight). Brain water content was calculated as a percentage via the following formula: ((wet weight—dry weight)/wet weight) ×100%.

### Detection of malondialdehyde (MDA) and Nox2 concentrations, and superoxide dismutase (SOD) and catalase (CAT) activity in the hippocampus

Four hours after reperfusion, the left hippocampal tissue each experimental group (n = 8) was homogenized on ice with normal saline, frozen in a refrigerator at -20°C for 5 min and centrifuged for 15min at 800 ×*g*. Supernatants were transferred into fresh tubes and the concentration of MDA production, as assessed by lipid peroxidation was measured using chemical assay kits (Nanjing Jiancheng Biologic Product). Results were expressed as nanomoles per milligram protein (nM/mg). The Nox2 ELISA kit (Blue Gene Biologic Product), a competitive enzyme immunoassay technique utilizing a monoclonal anti-Nox2 antibody and a Nox2-HRP conjugate was used to determine Nox2 concentrations. The results were expressed as above. SOD and CAT activity were measured using chemical assay kits (Nanjing Jiancheng Biologic Product) and results were expressed as activity unit per milligram protein.

### Detection of Tumor Necrosis Factor-α (TNF-α) and Interleukin (IL)-6 in Serum

A total amount of 3 mL of blood was drawn from each group (n = 8) 4h after reperfusion. Serum concentrations of TNF-α and IL-6 were determined using commercially available ELISA kits (Blue Gene Biologic Product) according to the manufacturer’s instructions and as described previously [25]. The results were expressed as picograms per milliliter serum (pg/mL).

### Statistical analysis

Statistical analyses were performed using SAS (version 9.13, SAS Institute Inc., Cary, NC, USA). Data were expressed as mean ± standard deviation (SD). Physiological parameters, blood glucose levels and arterial blood gas levels were analyzed using repeated measures of two-way analysis of variance (ANOVA) followed by Tukey’s post-hoc test. All other experiments were assessed by two-way ANOVA followed by post-hoc test. Bivariate Correlations with Spearman was used to analyze the correlation between the levels of post-ischemic inflammation or oxidative stress and apoptosis. All *p* values are two-tailed and a *p* value < 0.05 was considered as significant for all statistical analyses in this study.

## Results

### Characteristics of diabetic animal model

Baseline body weight and blood glucose levels were similar in all groups at the time just prior to STZ injection. As shown in **Table 1**, 4 weeks after STZ injection the diabetic rats displayed weight loss compared with non-diabetic rats (*p* < 0.05). At this time point, the levels of blood glucose remained normal in all nondiabetic rats. However, the blood glucose levels were significantly higher in diabetic rats than non-diabetic rats (*p* < 0.05).

**Table 1:** The Characteristics and physiological variables of rats in the experimental groups.

	NGC (n = 16)	NGD (n = 16)	HGC (n = 16)	HGD (n = 16)
**Body weight (g)**				
**Before STZ/buffer injection**	289.3±11.1	302.7±6.3	299.2±11.2	298.3±10.1
**4 weeks after injection**	286.3±13.4	311.8±15.4	209.2±27.3*	210.1±30.2*
**Blood glucose (mmol/L)**				
**4 weeks after injection**	4.9±0.8	5.2±1.2	17.8±4.7*	17.6±5.2*
**30 mins after reperfusion**	5.5±1.1	5.7±2.0	20.1±5.1*	18.9±6.1*
**MAP (mmHg)**				
**Before ischemia**	91.3±10.4	89.1±17.5	98.3±11.8	88.2±14.3
**After ischemia**	35.1±1.3^#^	36.2±2.7^#^	36.4±3.1^#^	34.4±1.1^#^
**30 mins after reperfusion**	111.1±8.2**	101.4±16.2**	98.3±16.3**	92.1±12.2**
**HR (rates/min)**				
**Before ischemia**	316.2±19.2	285.3±42.3	287.4±50.3	279.1±20.2
**After ischemia**	316.1±24.6	295.3±32.2	268.6±42.3	263.1±40.4
**30 mins after reperfusion**	314.1±13.3	303.4±37.1	286.5±47.2	308.3±32.3
**pH**				
**Before ischemia**	7.39±0.01	7.41±0.02	7.41±0.02	7.42±0.03
**30 mins after reperfusion**	7.37±0.04	7.39±0.05	7.39±0.03	7.38±0.04
**PaCO**_**2**_ **(mmHg)**				
**Before ischemia**	38.2±3.1	37.5±2.8	39.2±2.9	38.1±2.3
**30 mins after reperfusion**	39.8±3.9	40.3±6.1	40.2±4.7	39.2±3.6
**PaO**_**2**_ **(mmHg)**				
**Before ischemia**	243.5±27.1	240.1±45.9	243.2±34.7	230.2±70.7
**30 mins after reperfusion**	237.4±40.9	242.1±39.5	236.4±50.2	239.5±70.2

* P < 0.05 vs. NGC and NGD

# P < 0.05 vs. the values before ischemia

** P < 0.05 vs. the values after ischemia. MAP, mean arterial pressure; HR, heart rate; PaO_2_, arterial oxygen tension; PaCO_2_, arterial carbon dioxide tension; PH, potential of hydrogen.

### Hemodynamic variables and physiological data

Hemodynamic and physiological variables, including mean arterial pressure (MAP) and heart rate (HR) were monitored before and during the cerebral ischemia, and after reperfusion. Arterial blood gas was monitored before the cerebral ischemia and after reperfusion. After ischemia and reperfusion, there were no differences among groups in MAP, HR, arterial oxygen tension (PaO_2_), arterial blood carbon dioxide tension (PaCO_2_), or pH. The blood glucose levels after reperfusion were also monitored, and there were no differences among the groups compared with the levels before ischemia (**Table 1)**. Cortical CBF was analyzed before, during, and after cerebral ischemia with results indicating that CBF was decreased in all groups and increased after reperfusion (**Fig 1B)**.

### Effects of dexmedetomidine on neuronal injury in normal and diabetic rats

**Fig 2:**
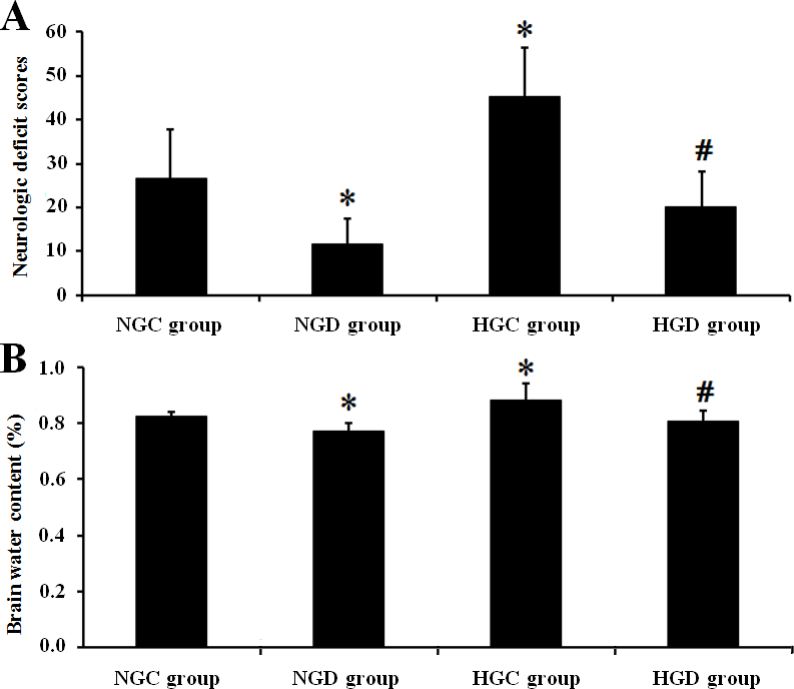
A, Neurologic deficit scores. The average scores for each rat for the 3 day scoring period are shown. Diabetes significantly increased the neurologic deficit scores compared with normal rats; dexmedetomidine decreased the scores in normal and diabetic rats. B, Brain water content in representative frontal and parietal lobes of each group. Diabetes significantly increased the brain water content compared with normal rats; dexmedetomidine decreased the content in normal and diabetic rats. * *p* < 0.05, ischemia vs baseline within all groups; # *p* < 0.05 reperfusion vs ischemia within all groups.

**Fig 3:**
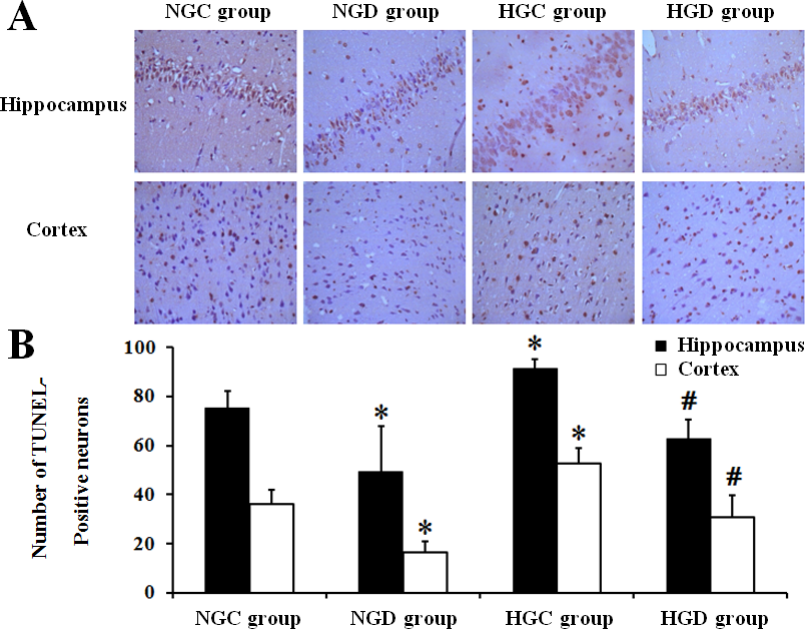
A, Immunohistochemical images of TdT-mediated dUTP nick end-labeling (TUNEL, 400×) in ischemic hippocampus and cortex 72h after I/R. TUNEL-positive cells significantly increased in diabetic rats compared with normal rats; dexmedetomidine significantly decreased the number of TUNEL-positive neurons in normal and diabetic rats. B, TUNEL-positive cell counts 72h after reperfusion. * *p* < 0.05 vs NGC group, # *p* < 0.05 vs HGC group.

Neurological scores (**Fig 2A**) and brain water content (**Fig 2B**) were significantly higher in diabetic rats compared with normal rats after I/R, and dexmedetomidine decreased the brain water content and the neurological scores in both normal and diabetic rats. TUNEL-positive cell counts indicated that compared with the NGC group, the HGC group showed significantly more TUNEL-positive cells in the hippocampus and cortex (*p* < 0.01), and dexmedetomidine reduced the TUNEL-positive cells significantly in the NGD group compared with the NGC group as well as in the HGD group compared with the HGC group (*p* < 0.01; **Fig 3A and 3B**).

**Fig 4:**
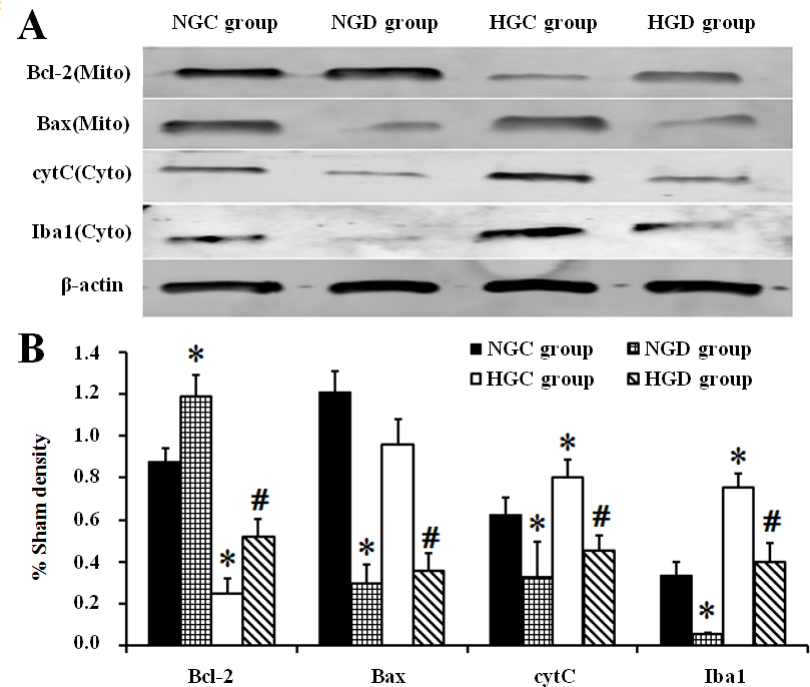
The concentration of apoptosis-related proteins and Iba1 in hippocampal tissue isolated from each group. A, Representative western blot bands of Bcl-2 (26kDa), Bax (20kDa), cytochrome c (11 kDa), Iba1(17kDa) and β-actin (43kDa) in the left hippocampus 4h after reperfusion. B, Quantification of western blot bands compared with β-actin (100%). * *p* < 0.05 vs NGC group, # *p* < 0.05 vs HGC group.

Western blotting demonstrated that both cytochrome c (Cyto) and Iba1 (Cyto) levels increased significantly in diabetic rats compared with normal rats after I/R (*p* < 0.05), and this increase was prevented significantly by dexmedetomidine treatment in both normal and diabetic rats t (*p* < 0.05). No increases were seen in Bax levels in the HGC group compared with the NGC group, but dexmedetomidine decreased Bax (Mito) levels in both normal and diabetic rats (*p* < 0.05). Bcl-2 (Mito) levels decreased significantly in diabetic rats compared with normal rats after I/R (*p* < 0.05) and this decrease was prevented significantly by dexmedetomidine treatment in both normal and diabetic rats (*p* < 0.05); **Fig 4**).

### Dexmedetomidine reduced oxidative stress in normal and diabetic rats

**Fig 5:**
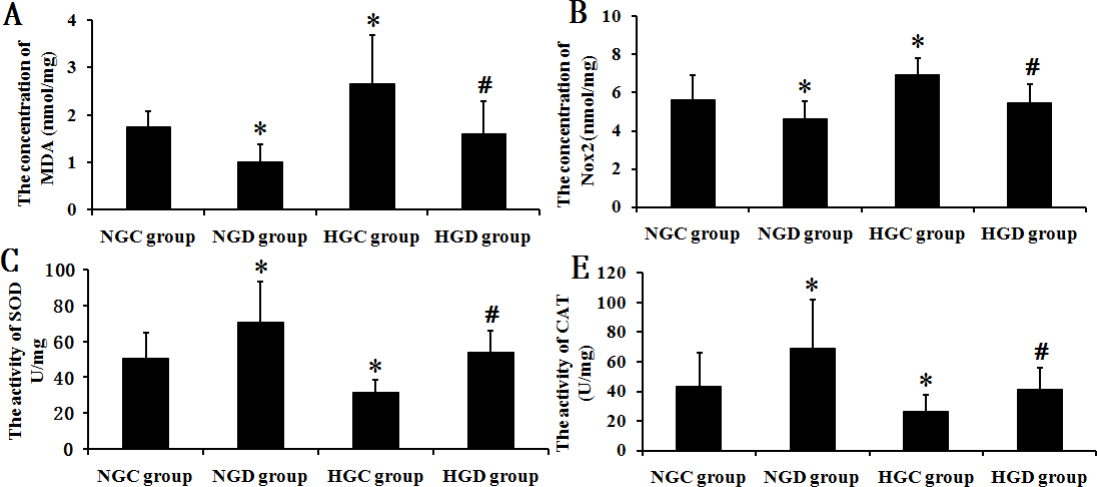
MDA and Nox2 levels along with, and SOD and CAT activity in hippocampal tissue isolated from each group. * *p* < 0.05 vs NGC group, # *p* < 0.05 vs HGC group.

As shown in **Fig 5A**, MDA levels increased significantly in diabetic rats compared with normal rats after I/R (*p* < 0.01), and this increase was prevented significantly by dexmedetomidine treatment when compared to each control group, respectively (*p* < 0.01). As shown in **Fig 5B,** Nox2 levels increased significantly in the HGC group compared with the NGC group (*p* < 0.01). Nox2 levels decreased significantly with dexmedetomidine treatment when compared to each control group, respectively (*p* < 0.01). Activities of SOD (**Fig 5C**) and CAT (**Fig 5D**) decreased significantly in the HGC group compared with the NGC group (*p* < 0.05), and dexmedetomidine increased the activities of SOD and CAT in both normal and diabetic rats (*p* < 0.05).

### Dexmedetomidine reduced plasma cytokine concentrations

**Fig 6:**
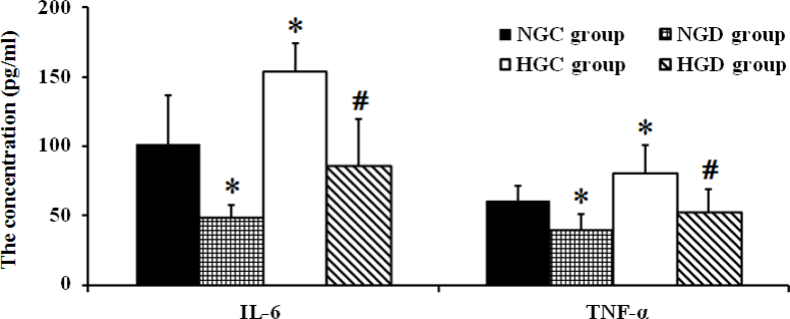
IL-6 and TNF-α serum concentrations in each group. * *p* < 0.05 vs NGC group, # *p* < 0.05 vs HGC group.

As shown in **Fig 6**, TNF-α and IL-6 levels increased significantly in the HGC group compared with the NGC group (*p* < 0.01), and these levels decreased significantly with dexmedetomidine treatment when compared to each control group, respectively (*p* < 0.01).

### Correlations between post-ischemic inflammation or oxidative stress and the apoptotic levels of neurons in the hippocampus and cortex

Significant positive correlations between Nox2, MDA, IL-6, TNF-α and apoptotic levels of neurons in the hippocampus and cortex were found. In addition, negative correlations between CAT, SOD and apoptotic levels of neurons in these regions were also found (**Table 2**).

**Table 2:** Correlations analysis results.

	Apoptosis of neurons	
	Hippocampus (r, *p*)	Cortex (r, p)
**NOX2**	0.538, 0.001	0.655, < 0.001
**CAT**	-0.520, 0.002	-0.485, 0.005
**SOD**	-0.418, 0.017	-0.612, < 0.001
**MDA**	0.512, 0.003	0.441, 0.012
**IL-6**	0.652, < 0.001	0.583, < 0.001
**TNF**	0.670, < 0.001	0.433, 0.013

NOX2, NADPH oxidases; CAT, catalase; SOD, superoxide dismutase; MDA, malondialdehyde; IL-6, interleukin-6; TNF, tumor necrosis factor-α.

## Discussion

In this study, we found that diabetes increased brain water content, the number of apoptotic neurons and early neurological deficit scores after transient global I/R. Intravenous injection of 5μg/kg/h dexmedetomidine for 90min was shown to significantly alleviate this damage in both normal and diabetic rats. The mechanism may be related to a reduction in both oxidative stress and inflammation during the initial cerebral I/R.

Our results showed that dexmedetomidine treatment upregulated the expression of the anti-apoptotic protein, Bcl-2 (Mito), and inhibited the expression of pro-apoptotic protein, Bax (Mito). By regulating the balance of anti- and pro-apoptotic proteins, dexmedetomidine decreased the subsequent release of cytochrome c into the cytosol in the present study. These results are partially consistent with a previous study, in which the administration of dexmedetomidine increased the concentration of Bcl-2 [18]. The release of mitochondrial cytochrome c is known to play a key role in the initiation of apoptosis [26], and this may explain the anti-apoptotic effects of dexmedetomidine in our study.

Oxidative stress plays an important role in the development of injury following I/R [27] and these effects are known to be exacerbated in diabetes [28]. Cerebral ischemia induces increased reactive oxygen species (ROS) production, such as O_2_^−^, H_2_O_2_ and NO. ROS production is further stimulated by reperfusion which causes cytotoxicity through lipid peroxidation, oxidation of proteins and DNA fragmentation [29, 30]. MDA is a low-molecular-weight aldehyde, originating from fragmentation of lipid peroxide radicals, and is an indicator for oxidative stress and lipid peroxidation [31]. In the present study, MDA levels in the HGC group were significantly increased compared with the NGC group, indicating that oxidative stress was enhanced in diabetic rats after I/R and furthermore, these levels were reduced after administration of dexmedetomidine. These results are partially consistent with a previous study in which researchers found that dexmedetomidine inhibited lipid peroxidation in normal glycemic rats [32]. Interestingly, similar effects were also found in hyperglycemia diabetic rats in the present study.

Recently sources of ROS generation have been targeted using antioxidant strategies. It has been established that there are three distinct mechanisms which generate ROS in neurons and contribute to cell death during anoxia and reoxygenation. Among the three, the third mechanism of ROS generation, initiated during reoxygenation, is predominantly caused by NADPH oxidases, including Nox2 [33]. Several studies have confirmed the neuroprotective effects of genetic deletion of Nox2 after cerebral I/R and strongly support the important role of Nox2 in stroke related oxidative stress damage. Lesion volume, edema formation, as well as early blood–brain barrier leakage were dramatically attenuated in mice lacking a functional Nox2 protein after stroke [34, 35]. Besides involvement during and after I/R, oxidative stress is also known to have a role in the etiology of diabetes [5]. Mitochondria are one of the known cellular compartments where the oxidative stress reaction originates. However, a previous study indicated that STZ-induced type I diabetes did not promote brain mitochondrial dysfunction, suggesting that oxidative stress associated with type 1 diabetes was not directly related to mitochondrial dysfunction, but more likely to extramitochondrial factors [36]. Evidence suggests that Nox2 is the principal source for cellular ROS in diabetes and could potentially be a therapeutic target for the treatment of diabetes [37]. Nox2 deficiency decreases β-cell destruction and preserves islet function in STZ-induced diabetes by reducing ROS production [38]. Therefore, the diabetic rats in our study suffered from double oxidative stress injury: one was from diabetes and the other from I/R with results indicating that Nox2 may play an important role in both these processes. In our study, Nox2 levels in the HGC group significantly increased compared with the NGC group. Administration of dexmedetomidine significantly inhibited the increased Nox2 in normal and diabetic rats. Due to the important role of Nox2 in oxidative stress during diabetes and I/R, we speculate that Nox2 inhibition is the main mechanism by which dexmedetomidine exerts protection from oxidative stress during I/R in diabetic rats. In brain tissue, microglia are primary cells expressing Nox2 [39]. Western blot analysis of Iba1 (Cyto) expression indicated that activated microglia were likely to be the main resource of the increased Nox2, therefore, the effects of dexmedetomidine on Nox2 may be related to inhibition of microglia.

Excessive ROS release leads to brain tissue damage during I/R. When ROS are generated, the defensive system of antioxidant enzymes, such as SOD and CAT, play an important role in the resistance of neurons from ROS-induced cell death [40]. SOD specifically detoxifies O_2_^−^ to H_2_O_2_, which is then scavenged by CAT. During I/R, H_2_O_2_ cannot be readily scavenged because of low activities of SOD and CAT [40]. A previous study indicated that dexmedetomidine increased the endogenous antioxidant defense enzymes and inhibited lipid peroxidation in hippocampus [32]. In the present study, results indicated that the antioxidant effect of dexmedetomidine was still evident, even in diabetic rats. Our study found that after I/R, dexmedetomidine inhibited the expression of Nox2 and decreased the production of ROS in both normal and diabetic rats. Meanwhile, there was an increase in SOD and CAT activity and an accelerated scavenging of ROS.

A previous study demonstrated that both inflammation and oxidative stress are involved in the process of I/R injury in an experimental model of diabetes [5] and increased pro-inflammatory cytokines including TNF-α and IL-6 were observed at early stages in transient global ischemia [41]. TNF-α predominately mediates immune and inflammatory responses [42] leading to apoptosis. The current data showed that dexmedetomidine reduced serum concentrations of TNF-α and IL-6 in normal and diabetic rats, which suggested that dexmedetomidine could confer a neuroprotective effect by inhibiting the inflammatory response. Several studies have demonstrated that dexmedetomidine may decrease the serum concentration of TNF-α after I/R injury [11, 32], and an *in vitro* study also showed that the α_2_ adrenergic receptor agonist suppressed IL-6 production [43]. Interestingly, the present study found that the anti-inflammatory effects of dexmedetomidine were still apparent in diabetic rats. We speculated this may be partially due to the suppression of Nox2. Indeed, a study indicated that Nox2 deficiency could attenuate the inflammatory response by decreasing induction of intercellular adhesion molecule-1 thus reducing neutrophil infiltration into the brain after I/R. A subsequent study from the same group confirmed the neuroprotective effects of Nox2 deletion in mice after I/R injury and also observed an attenuation of post ischemic inflammatory gene expression [44].

Accumulating evidence has affirmed the neuroprotective role of dexmedetomidine, and the α_2_ adrenergic receptor is regarded as the target receptor [17, 18, 45]. There is a growing body of evidence indicating a direct link between diabetes and detrimental effects in the central nervous system [46–48], and many studies have found central noradrenergic processes are altered in diabetes. The sleep time induced with dexmedetomidine was significantly shortened when tested 10 days, 3, 6 and 8 weeks into the diabetic state [49]. Zhang reported that the central diuretic and natriuretic effects of the α_2_ adrenergic receptor agonist, clonidine, have been suppressed in STZ-treated rats [50]. The attenuation of the anti-nociceptive effects of clonidine were observed following systemic and intrathecal administration of the agonist in diabetic animals [51]. These data suggested that the impaired responsiveness of diabetic rats might be due to a central α_2_ adrenoceptor desensitization and/or biochemical defects in the post receptor response. However, to date, whether the neuroprotective effects of dexmedetomidine are altered remain unclear. Our study suggested that the neuroprotective effects of dexmedetomidine are still evident in diabetic animals. We speculated that two factors may be involved in this finding. First, the α_2_ adrenergic receptor may still play a part in the neuroprotective effects of dexmedetomidine, although the function and levels of the receptor were possibly altered. Second, *in vitro* and *in vivo* studies indicated that dexmedetomidine can exhibit properties against oxygen and glucose deprivation-induced (OGD) injury or I/R injury, independent of the α_2_ adrenergic receptor, possibly through imidazoline receptors [52, 53]. Furthermore, some studies suggested that the properties of dexmedetomidine against OGD injury are related to the imidazoline I_1_ receptors-extracellular-regulated kinases pathways, and this process is likely independent of the α_2_ adrenergic receptor response [52, 54]. Another study found that dexmedetomidine protection against OGD injury was predominantly blocked by the imidazoline I_2_ receptor antagonists, idazoxan and BU 224, but not by the α_2_ adrenergic receptor antagonist, yohimbine, indicating that imidazoline I_2_ receptors have a role in this method of protection [55]. Another *in vitro* study indicated that imidazolines exert a strong neuroprotective effect against excitotoxicity and hypoxia in cerebellar and striatal primary neuronal cultures by inhibiting NMDA receptors [56]. As NMDA receptors have an important role in Nox2 activity and oxidative stress [57, 58], we speculated that in our study the decreases in Nox2 and the neuroprotective effects of dexmedetomidine in diabetic rats may be partly regulated by the imidazoline receptor-NMDA-Nox2-oxidative stress pathway.

According to the body surface area normalization method which was recommended by the Food and Drug Administration [59], the dose of dexmedetomidine used before the ischemic insult in rats in our experiment was equivalent to an approximate dose of 0.8 μg/kg/h in humans. The guidelines for this method and the dose used in this study closely mimics that which is found in human clinical practice. We found that dexmedetomidine administered at the above dose can protect the brain after I/R in both normal and diabetic rats. These findings suggest that dexmedetomidine may also be used perioperativly as an adjunct with optimal anesthetic management for diabetic patients with high cerebral ischemic risk.

There are limitations of the present study. We found dexmedetomidine inhibited Nox2 and subsequent oxidative stress, but further studies are needed to confirm which receptor pathways are affected by dexmedetomidine administration. We speculated that microglia were the main source of the increased Nox2 because of the increased Iba1 expression in our study. Again further studies are need to confirm this hypothesis as well as is the need to test for the generation of endogenous ROS and inflammatory proteins.

## Conclusion

In conclusion, we highlighted the neuroprotective effects of dexmedetomidine in diabetic animals, which prove clinically beneficial for patients with diabetes.
